# The Acquisition of Survey Knowledge by Individuals With Down Syndrome

**DOI:** 10.3389/fnhum.2020.00256

**Published:** 2020-07-03

**Authors:** Zachary M. Himmelberger, Edward C. Merrill, Frances A. Conners, Beverly Roskos, Yingying Yang, Trent Robinson

**Affiliations:** ^1^Division of Behavioral Sciences, Maryville College, Maryville, TN, United States; ^2^Department of Psychology, The University of Alabama, Tuscaloosa, AL, United States; ^3^Department of Psychology, Montclair State University, Montclair, NJ, United States

**Keywords:** down syndrome, survey-knowledge, landmark memory, spatial abilities, MA comparison

## Abstract

People with Down syndrome often exhibit deficiencies in wayfinding activities, particularly route learning (e.g., Courbois et al., 2013; Davis et al., 2014; Farran et al., 2015). Evidence concerning more sophisticated survey learning has been sparse. In the research reported here, two experiments are reported that evaluated survey learning of youth with DS and typically developing children (TD) matched on mental age. In Experiment 1, participants learned two overlapping routes consisting of three turns each through a virtual environment depicting 9 square city blocks. Following acquisition, they were tested on multiple measures of survey knowledge: finding a shortcut, identifying the direction of landmarks not currently visible from their location in the environment, and recognizing a bird’s-eye representation of the overall environment. Under these conditions, which should provide relatively optimal opportunities for survey learning, the participants with DS performed comparably to TD participants matched on non-verbal ability on all of our measures of survey learning. Hence, we concluded that people with DS can acquire some survey knowledge when tasked with learning a small environment and given the opportunity to do so. In Experiment 2, the experimenter navigated participants through a large, relatively complex, virtual environment along a circuitous path, beginning and ending at a target landmark. Then, the participants were placed at a pre-specified location in the environment that they had viewed previously and instructed to navigate to the same target (a door) using the shortest possible path from their current location. They completed the task three times: once after being shown the environment one time, once after three exposures, and once after five exposures. Results indicated that the participants with DS exhibited significantly less skill at identifying the shortcut than did the TD participants, with differences emerging as the number of exposures increased. Participants with DS were also less able to recall landmarks at the end of the experiment. Overall, however, the performance of both groups was relatively poor in both experiments – with the performance of participants with DS being worse as conditions became less optimal. These results were discussed in terms of underlying mechanisms that may account for variations in survey learning as environmental complexity increases.

## Introduction

Down syndrome (DS) results from the presence of a full or partial copy of extra chromosomal material associated with chromosome 21. It is the most common genetic syndrome associated with Intellectual Disability (ID) (Dykens et al., 2000), with a prevalence reported at 1 in 700 births (Parker et al., 2010). The expression of DS includes physical, cognitive, and neuroanatomical abnormalities. Characteristic physical features may include poor overall muscle tone, flattened facial features, upward slanting eyes, wide short hands and fingers, small head and ears, and a protruding tongue (Bull and The Committee On Genetics, 2011). Cognitive impairments in DS include well documented deficits in speech and language (e.g., Fowler, 1990; Martin et al., 2009) and problems with verbal short-term and long-term memory (e.g., Wang and Bellugi, 1994; Jarrold et al., 1999; Godfrey and Lee, 2018). More recently, evidence has been presented suggesting that some aspects of visuo-spatial processing may also be impaired in individuals with DS (see Yang et al., 2014). In particular, researchers have identified poor performance with mental rotation (Meneghetti et al., 2018), the acquisition of visuo-spatial knowledge (Meneghetti et al., 2017), and the use of navigation and wayfinding skills (e.g., Courbois et al., 2013; Davis et al., 2014; Farran et al., 2015). Neuroanatomically, DS is characterized by smaller brain volumes, particularly associated with the cerebellum, frontal lobes, and temporal lobes (e.g., Pinter et al., 2001b; White et al., 2003; Dierssen, 2012). In addition, studies have reported smaller volumes for the hippocampus and corpus callosum (Aylward et al., 1999; Pinter et al., 2001a), as well as the entorhinal cortex (Dierssen, 2012; Guidi et al., 2018). Longitudinal differences in hippocampal volume have been associated with decreased cognitive functioning (Pujol et al., 2018).

There is considerable overlap between brain regions that are impacted by DS and those regions that support wayfinding/navigation activities. Wayfinding is generally thought to involve a fairly distributed network of brain regions (Boccia et al., 2014). The temporal lobes appear to play an important role in memory-guided navigation (Pine et al., 2002). The cerebellum has recently been identified as contributing to both motor and cognitive aspects of navigation (e.g., Iglói et al., 2015). Further, because navigation is a goal-oriented activity, evidence indicates a necessary role for frontal lobes in navigation (Ciaramelli, 2008) which may be related to the ability to keep the goal in mind during navigation activities and making navigation plans (Spiers, 2008).

There is ample neuroscientific and behavioral evidence that wayfinding depends heavily on two distinct but related mental representations, often termed route and survey knowledge. Route representations involve ordered connections of landmarks, whereas survey knowledge is a more sophisticated and flexible form of environmental representation that involves acquiring knowledge of the directions and relative distances between objects and locations within an environment that is independent of any specific route (Siegel and White, 1975), and is related to Tolman’s (1948) conception of a cognitive map. Spatial relational processing, which is important for developing survey knowledge, seems to rely on a distributed network in the hippocampal region. The hippocampus proper is known to play a prominent role in the learning and memory of novel and recently learned environments (e.g., Claessen et al., 2019), responsible for considerable spatial relational processing (Kumaran and Maguire, 2005), and engaged in the learning of survey representations, which provide a mental representation of the physical environment (Schinazi et al., 2013). Specifically, the left hippocampus seems to be important for encoding relations between landmarks (Wolbers and Büchel, 2005), whereas the right hippocampus is associated with retrieval of relational information (Mellet et al., 2000). At the cellular level, place cells in the hippocampus provide a mechanism for encoding relative spatial location (O’Keefe and Nadel, 1978), which can be updated through movement and head direction (see Burgess, 2008). A full path integration model can be developed at the cellular level when also including the medial entorhinal cortex (McNaughton et al., 2006). Given neuroanatomical abnormalities and associated cognitive weaknesses that people with DS exhibit related to the hippocampus, it would not be at all surprising to find that people with DS would exhibit difficulties with wayfinding.

A small number of studies have supported the view that people with DS exhibit relatively poor wayfinding skills. Several of these studies focused on the acquisition of route knowledge, which involves the acquisition and memory of a fixed sequence of landmarks and turns to get from a starting location to a designated target location (Siegel and White, 1975). For example, Purser et al. (2015) examined route learning in adolescents and young adults with DS relative to typically developing (TD) children and participants with Williams Syndrome (WS). They found that their participants with DS could learn a six-turn route. However, the performance of the participants with DS depended on their non-verbal ability level. Participants with DS who were relatively low in non-verbal ability performed below that of the TD participants, whereas those who exhibited higher levels of non-verbal ability performed at levels similar to TD participants. Davis et al. (2014) reported two studies that indicated adolescents and young adults with DS exhibited more errors during route learning and took longer to learn routes than did a comparison group of participants with mixed etiology ID and a group of typically developing children with whom they were matched on non-verbal mental age. Farran et al. (2015) assessed route learning of adolescents and young adults with DS relative to TD children and adolescents and young adults with WS. They found that the participants with DS committed more errors during acquisition of two overlapping routes than did the TD children, although they performed similarly to the participants with WS.

Courbois et al. (2013) and Farran et al. (2015) evaluated the ability of people with DS to form shortcuts following the learning of two short routes. Courbois et al. (2013) reported that only two of seven participants with DS were able to identify the shortest path along two overlapping routes to find a target from the start of one learned route to the end of the second learned route. However, five of nine TD children matched on mental age (MA) were able to do so. Similarly, Farran et al. (2015) reported that only 10% of participants with DS were able to identify the shortest route using a novel path along across two previously learned routes, whereas 59% of their TD children found the shortest route. Hence, it appears that the acquisition of survey knowledge may also be a problem for people with DS. However, this conclusion is based on two studies that have focused on one aspect of survey knowledge, the ability to identify a shortcut that was not explicitly taught. One additional study has been reported that involved presenting survey knowledge in the form of sketch maps to assist environmental learning (Meneghetti et al., 2017). These researchers found that although their participants with DS benefited from the presence of the maps they did so to a lesser degree than did TD children. In our study, we expand upon the available research by looking at the learning of survey knowledge following different levels of exposure to the environment and evaluating multiple aspects of survey knowledge.

One goal of the current investigation was to replicate and extend our understanding of survey learning performance of people with DS. Hence, we examined performance using multiple measures of survey knowledge under optimal conditions of environmental learning, where participants were required to learn two routes through a virtual environment prior to assessing survey knowledge. For Experiment 1 we selected a relatively small, predictable environment (i.e., 9 square city blocks). We then provided sufficient exposure that allowed all participants to learn two overlapping routes that traversed the full environment. Finally, we evaluated several different measures of survey knowledge after the two routes had been sufficiently learned. More specifically, whereas previous research conducted on people with DS have used finding shortcuts as the primary measure of survey knowledge, there are several additional ways that knowledge of the environment can be assessed. For example, survey knowledge is demonstrated when making direction or distance estimates and identifying maps of the overall environment, in addition to finding the most efficient route to a target (Blades, 1997; Montello, 1998).

The use of additional measures of survey knowledge may provide greater insight into the kind of survey knowledge encoded by people with DS. More specifically, finding a shortcut requires that individuals recognize similarities across routes that they directly encountered. Direction estimation requires a more abstract representation that requires an understanding of relations between objects in the environment that were not directly perceived. Choosing a bird’s eye view map representation of the overall environment requires fully integrating the segments into a coherent whole over multiple experiences. Therefore, to assess survey knowledge we asked participants to find a shortcut, identify the direction of landmarks not currently visible from their location in the environment, and identify a bird’s eye representation of the overall environment. Given the conditions of Experiment 1, the results would be expected to reveal whether or not participants with DS have the basic capacity to acquire survey knowledge and use that knowledge to navigate a small, predictable environment. If participants are better at using some aspects of survey knowledge relative to others, it may be possible to identify specific mechanisms and strategies that operate differently during survey learning for people with DS.

A second goal of the current investigation was to identify differences in the acquisition of survey knowledge across multiple exposures to the environment in TD children and people with DS. We were specifically interested in whether differences emerged under less optimal conditions of learning. Therefore, we created conditions that were less optimal for environmental learning in Experiment 2. The environment was both more complex and less predictable in overall layout in the second study. Our evaluation of ongoing learning was prompted by recent studies indicating that learning of survey knowledge can begin with initial exposures to the environment. Although early perspectives of wayfinding suggested that survey knowledge develops from route knowledge (e.g., Siegel and White, 1975), Montello (1998) has argued that survey representations develop more gradually and become more accurate over repeated exposures to novel environments rather than in distinct stages. Ishikawa and Montello (2006) demonstrated that individuals can develop at least some survey knowledge at their first exposure to a new environment. In that study, the researchers drove participants through two novel large-scale environments once a week for 10 weeks. The routes were connected but participants were not made aware of that connection until week 3. Participants learned the location of buildings on each route and were asked to complete several measures of survey knowledge, including pointing to the location of unseen buildings, making distance estimates, and drawing sketch maps of the environment. Ishikawa and Montello (2006) reported that individuals performed better than chance levels when sketching a map of the environment after their first exposure. This was interpreted to mean that some survey knowledge was being developed in conjunction with route learning, contrary to the strictly hierarchical view suggested by Siegel and White (1975). However, as indicated by the researchers, the use of heuristics may lead to better than chance performance, as many environments are structured similarly. Additionally, Buchner and Jansen-Osmann (2008) showed that distances between landmarks can support route learning (see also Latini-Corazzini et al., 2010). This provides some evidence that survey learning occurs concurrently with, rather than subsequent to, other kinds of environmental knowledge.

In Experiment 2, individuals with DS and a group of TD children approximately matched on non-verbal ability (MA) experienced an environment multiple times. Their ability to navigate the environment was examine after one, three, and five exposures. This allowed us to examine whether survey knowledge accrued at different rates for participants with DS relative to TD participants matched on non-verbal ability. Based on previous research (e.g., Davis et al., 2014) indicating a slower accrual of route knowledge by participants with DS, we expected that the participants with DS in Experiment 2 would exhibit slower acquisition of survey knowledge as well.

We also evaluated landmark learning in Experiment 2. Landmarks are integral to learning about the environment for navigation purposes (Presson and Montello, 1988). There has been a considerable amount of research demonstrating the benefit of landmarks to route learning and wayfinding (Walkowiak et al., 2015). However, the process of identifying landmarks is fairly complex. Research has shown that visibility, frequency among multiple contexts, and relation to decision points are all important factors influencing landmark selection (Caduff and Timpf, 2008). Further, specific positions of landmarks at intersections can influence the utility of the landmark for reproducing a path versus navigating an alternative route such as a return path (Karimpur et al., 2016; Balaban et al., 2017).

Although our focus was on shortcut finding relative to finding a return path, it is reasonable to expect that difficulties with perspective taking and selection of landmarks at locations more relevant to the original path participants are shown compared to the shortcut test may impede identifying a shortcut. Further, persons with DS who exhibit difficulties with executive function processes that are responsible for visual perspective taking (Rowe et al., 2006; Lanfranchi et al., 2010) may be expected to have greater difficulties choosing paths from alternative directions than TD children. This difficulty would be compounded by difficulties in remembering landmarks in general, as would be predicted based on Davis et al. (2014).

## Experiment 1

Experiment 1 was designed to investigate the type of survey knowledge acquired during route learning under relatively optimal conditions by people with DS compared to typically developing (TD) children with whom they were matched on level of intellectual functioning. Participants learned two routes through a simple, predictable virtual environment consisting of depictions of city streets. Once the routes were learned, they engaged in three new tasks that were presumed to assess different aspects of survey knowledge. First, they were asked to find a target in the environment using a shortcut route that they had not learned and was available only if they had integrated the two individual routes into a combined, connected representation of the environment. Second, they were asked to point to the location of an unseen object in the environment using straight line direction judgments. This measures the extent to which participants represented landmarks allocentrically – in relation to each other rather than in relation to the self – in their survey representation of the environment. Third, they were shown several depictions of the environment and asked to identify which depiction most accurately represented the environment they had just learned to navigate. This measures the extent to which their spatial knowledge includes an allocentric representation of the entire environment.

### Method

#### Participants

The participant groups consisted of 12 adolescents/young adults with DS and 12 TD children. Participants were paid $5.00 for completing the tasks. The participants with DS were recruited from the University of Alabama Intellectual Disabilities Participant Registry and from local service providers. Parents or guardians confirmed a diagnosis of DS. The TD participants were recruited from local preschool programs. The groups were approximately matched on KBIT-2 Matrices Raw scores. The participants with DS performed slightly better on the KBIT Matrices (21.0: *SD* = 9.2 vs. 19.8: *SD* = 3.9, for DS and TD, respectively), although this difference was not significant (see section “Results”). However, the participants with DS were clearly more variable in performance on the KBIT-2 than were the TD children. The mean age of the participants with DS was 19 years and 2 months (*SD* = 26 months) and for the TD children was 5 years and 0 months (*SD* = 4 months).

#### Measures

##### Kaufman brief intelligence test-2

Raw scores on the KBIT-2 Matrices subtest were used to match groups on non-verbal ability. The Matrices subtest consists of a 2 × 2 or 3 × 3 grid of pictures with one element missing. Participants are asked to choose which one of five pictures best completes the grid. The KBIT-2 was selected because it has good reliability [between 0.87 and 0.91 based on split-half and 0.76 and 0.89 based on test–retest reliability for TD participants in the age range tested and participants with Intellectual Disability (Kaufman and Kaufman, 2004)]. Further, it correlates well with the Leiter-R (*r* = 0.62) in children with special needs (e.g., Scattone et al., 2012).

##### Virtual environment task

**FIGURE 1:**
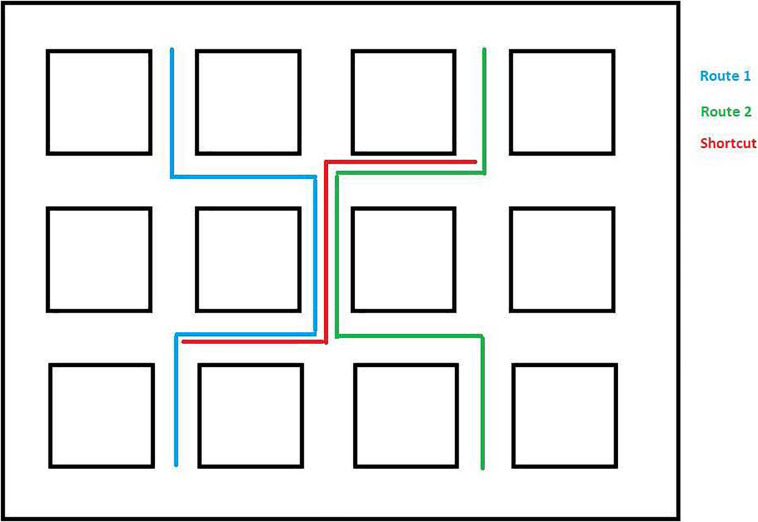
Overview of Experiment 1 environment with learned routes and shortcut route depicted.

**FIGURE 2:**
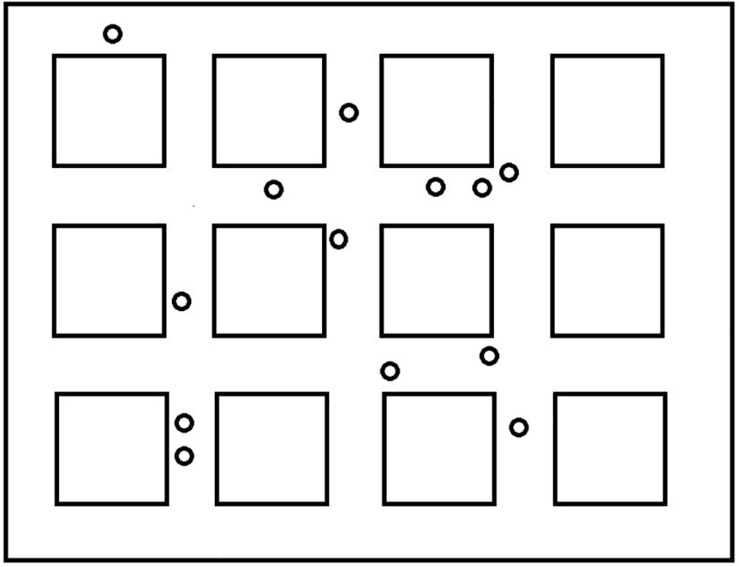
Locations of landmarks used during the direction-of landmarks task.

The virtual wayfinding task was created using the Valve Hammer editor version 4.1 and presented using Portal 2. The environment consisted of twelve square blocks along city streets. The environment was selected to be consistent with previous studies (e.g., Courbois et al., 2013), to allow for overlapping routes, and to ensure a reasonable likelihood that participants could learn the simple routes in a few exposures (see Figure 1). There were 13 landmarks spaced throughout the environment that participants would encounter along the routes to which they were exposed (see Figure 2). All of the landmarks were visible from the trained routes and were identified by the experimenter as they traveled the routes the first time. Within the environment we created two overlapping routes that included 4 turns each. These were the routes that participants were taught during the training phase. These are also shown in Figure 1. A third route, the Shortcut, was constructed to include one segment from Route 1 only, one segment from Route 2 only, and one segment used on both routes. One specific landmark, a green trash can, was located along Route 2 and was the target for the Shortcut route. This landmark was identified as an important landmark to remember as participants learned Route 2. All computer tasks in Experiment 1 were completed on a Dell Inspiron 7548 laptop with a 15.6″ monitor and a screen resolution of 1,920 × 1,080 pixels. All participants sat approximately 60–75 cm from the display.

##### Route learning errors

Participants received up to six trials to learn each route. Wayfinding errors were recorded for all trials. An error consisted of taking one step down an incorrect segment, whether by making a wrong turn or going straight when a turn was required. If participants made an error, they were verbally redirected back to the correct path by the experimenter to finish the trial. If participants completed the route successfully prior to making all six trials, they moved on to the next part of the procedure. However, we ultimately used only the number of errors across the first three trials for each route as our measures of wayfinding errors because more than half of the participants did not need the last three trials when learning Route 1 (7 participants in each group).

##### Shortcut navigation task

Following exposure to both routes, participants were placed at a location along Route 1 (see Figure 1), and asked to travel the shortest distance to get to the target (a green trash can) that was along Route 2. The target was specifically identified as the participants traveled Route 2 during training (see section “Procedure”) to ensure they knew what the target was and where it was located. They were given one attempt at finding the shortest route. The number of segments traveled to reach the target was used as the dependent measure. The shortest possible route was 3 segments long. Note that there was an alternative to the shortcut (see Figure 1) that was also three segments long that did not include the overlapping segment. However, none of our participants took that route. Hence, this possibility was not considered in the analysis.

##### Direction of landmarks

**FIGURE 3:**
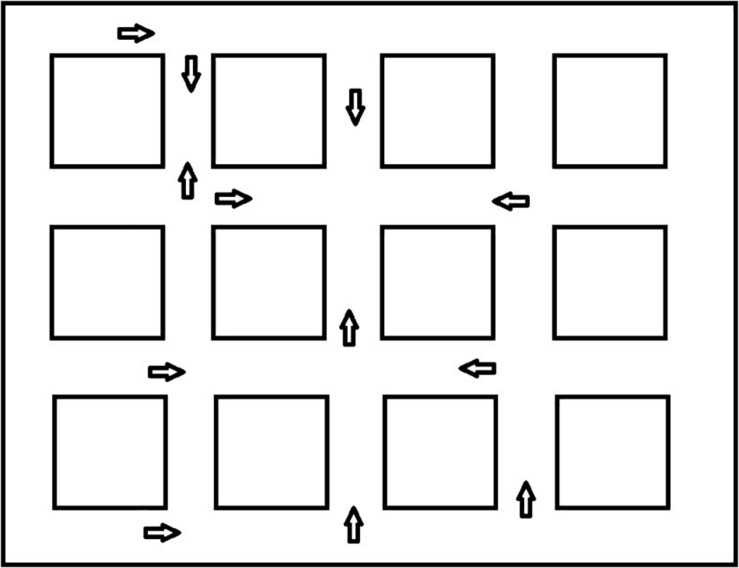
Pointing locations and facing directions for the landmark directions task.

**FIGURE 4:**
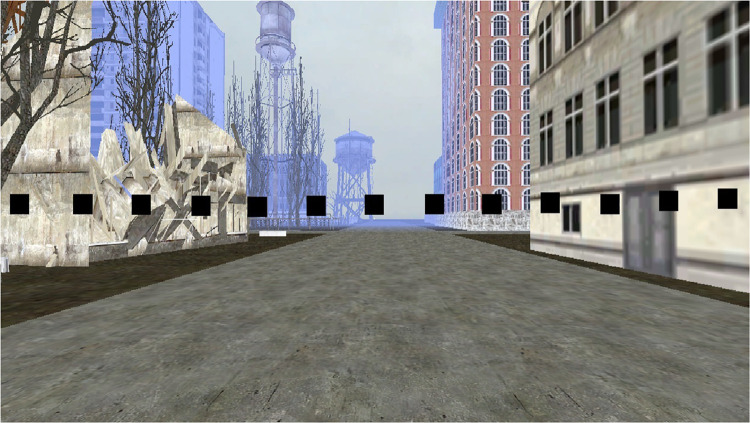
Participant view in the direction of landmarks task.

Following the shortcut task, participants were placed at different locations along one of the routes and asked to locate various landmarks. Figure 3 presents the different locations where the participant was placed in the environment. They were positioned to look straight ahead down a street and saw a scene that included 13 small black squares in a line across the screen (see Figure 4 for the participant’s view). The squares represented a range of approximately 45 degrees from the left to the right of the monitor. Squares were approximately 3.0 degrees of visual angle apart from each other from center to center. Participants were shown a picture of one of the landmarks and asked to point to the black square that was closest to where they thought the landmark would be from where they were standing, even if they could not see it. The first pointing trial was considered a practice trial and the landmark was visible from the participant’s location. After each trial, the participant was invisibly moved to a new location (we told them they were transported), viewed a new scene with squares, and shown a new landmark to locate. We recorded which square the participant indicated. This was repeated for all 12 remaining landmarks. We wanted to be sure that participants understood the basic pointing instructions and were not simply responding randomly. We reasoned that they should be more accurate for visible landmarks if this was the case. Therefore, during testing, four landmarks were visible to the participant and 8 were not visible to the participant. We recorded the square that was identified by the participants as the one closest to the landmark. The dependent measure was divergence from the actual location of the landmark in terms of visual angle (3.0 degrees for each square from the target landmark). Then we averaged across trials to calculate an average degree of divergence for each participant. In a given trial, it was possible for maximum pointing errors to range from 0 degrees (if the target object was directly behind the selected square) to 36 degrees of visual angle (if the target object was at either extreme right or left location and the participant chose the opposite extreme). However, in most trials, the maximum pointing error was less than 36 degrees because the correct square was closer than the edge of the screen. After averaging the trials, an error of 27 degrees was the maximum score and 0 degrees was the minimum score. Completely random responding would result in an average error of 13.5 degrees.

##### Map recognition task

**FIGURE 5:**
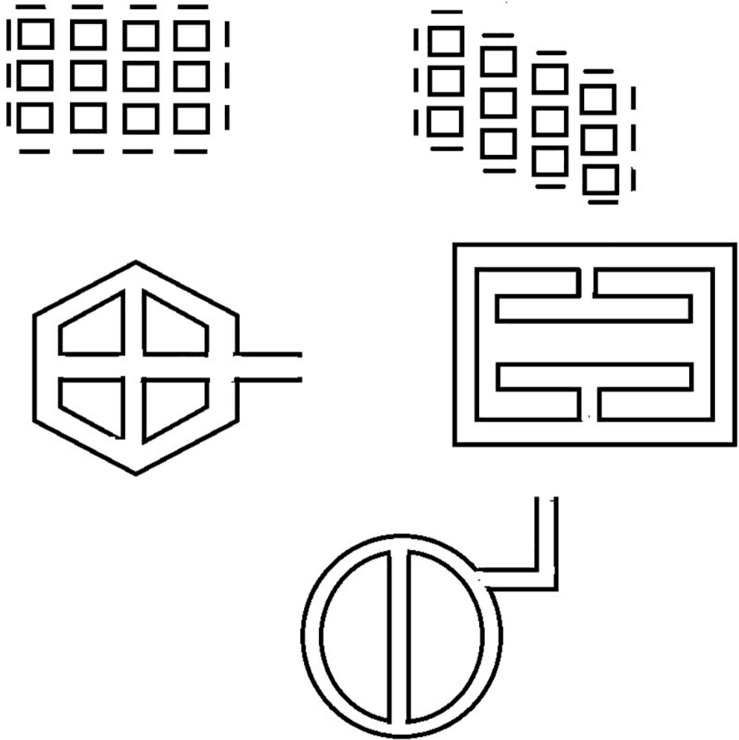
Stimuli for the map recognition task. Correct figure in the upper left.

For the last measure, participants were shown five depictions of a bird’s eye view of the environmental layout (see Figure 5). They were asked to select the picture that most closely resembled the layout they have been navigating. We recorded whether or not the participant selected the correct picture.

##### Procedure

Parents or guardians gave consent and all participants gave assent. All participants were first administered the Matrices Subtest of the KBIT-2. Then they were presented the virtual environment. Following familiarization with the mouse controls, they were trained on the two overlapping routes. During the training phase, participants were first shown training Route 1 by the experimenter. This was experimenter controlled, rather than presented in video format, to allow the experimenter to refocus the participant’s attention to the route if they appeared to look away during the initial viewing of the route. Then they were given three trials to retrace the path on their own. If they were unable to retrace the path after three attempts, they were shown Route 1 a second time and given three more trials to retrace the route. Following a maximum of six trials, they were switched to Route 2. However, if they were able to successfully retrace Route 1 without error, they were immediately switched to Route 2 without having to make all six attempts. Route 2 was presented in the same manner as Route 1. They were exposed to the route, given up to six trials to navigate the route successfully, then they were exposed to the route a second time if needed prior to achieving a successful attempt without error to a maximum of six trials. An error consisted of entering an incorrect segment. When an incorrect segment was entered during the route learning phase participants were told “This is not the way I went. Can you turn around and go the way I went?” this was repeated for all errors. The number of errors was recorded for each attempt. Landmarks used in the pointing task were identified as the participants were shown the routes. A green trash can was specifically identified as a landmark to remember when learning the second route. This was the target of the shortcut task.

Following a maximum of six trials to learn each of the two routes, the shortcut task was presented. Participants were placed at the beginning shortcut location on the first path (see Figure 1). They were told that “Someone dropped something important in the green trash can that you saw along the second path. Can you find the shortest way to the green trash can?” The number of segments traveled was recorded.

Following the shortcut task, participants completed the direction of landmarks task and the map recognition task in the order. The order of presentation was fixed to prevent participants from using information in the later tasks to perform the earlier tasks.

### Results

#### KBIT-2

Primary data for Experiment 1 are presented in Table 1. A preliminary analysis of MA scores revealed that groups were not significantly different on measured KBIT-2 Matrices raw scores [*F*(1,21) = 0.163, *p* = 0.690, $\eta_{\text{p}}^{2}$ = 0.008]. However, because of the variability in KBIT-2 scores between groups, all analyses performed using standard Analysis of Variance procedures were followed by Analysis of Covariance using KBIT-2 scores as a covariate. No differences between any statistical comparisons were found using ANCOVA relative to ANOVA. Hence, only ANOVA results are reported.

**TABLE 1:** Mean results from Experiment 1 for each Group and Task.

Group	Task^1^	Measure	Mean^2^
Down syndrome	Route learning (12)	Route 1 errors	
		Trial 1	2.4 (1.9)
		Trial 2	1.2 (1.4)
		Trial 3	2.6 (2.2)
		Route 2 errors	2.3 (4.0)
		Trial 1	1.5 (2.1)
		Trial 2	1.0 (1.2)
		Trial 3	2.0 (2.7)
	Shortcut (10)	Segments	4.5 (1.6)
	Direction of visible landmarks (10)	Degrees of divergence	7.5 (3.6)
	Direction of non-visible landmarks (10)	Degrees of divergence	8.4 (3.6)
	Map recognition (10)	# of participants choosing correctly	5
TD children			
	Route learning (12)	Route 1 errors	
		Trial 1	2.9 (2.2)
		Trial 2	1.9 (2.0)
		Trial 3	1.6 (1.4)
		Route 2 errors	
		Trial 1	1.4 (1.2)
		Trial 2	1.3 (1.6)
		Trial 3	1.7 (2.0)
	Shortcut (11)	Segments	6.8 (1.9)
	Direction of visible landmarks (12)	Degrees of divergence	7.5 (2.7)
	Direction of non-visible landmarks (12)	Degrees of divergence	11.7 (5.7)
	Map recognition (12)	# of participants choosing correct map	6

**Map recognition reflects number of participants who selected the correct map. ^1^Number of participants completing each Task in parentheses. ^2^Standard deviation in parentheses.**

#### Route Learning

Growth curve analysis was used to analyze the number of errors taken to find the target during the route learning task. This approach provides several benefits over a traditional repeated measures ANOVA for data that is clustered within an individual, including the ability to handle missing data and to account for individual variation in the statistical effects. For the purposes of this study, the main benefit of growth curve analysis is the ability to examine trends across the repeated trials. For a brief discussion of growth curve analysis, see Curran et al. (2010). A more detailed discussion can be found in Snijders and Bosker (2012).

We started with a basic model and then sequentially added the fixed effects and compared the new model to the previous model. The improvements on model fit were evaluated using -2 times the change in log-likelihood. Statistically significant improvements in model fit indicate that the model with the added variable better explains the observed results. If the change in model fit was not significant, we retained the original model. All model coefficients were estimated using maximum likelihood estimation using the lme4 (Bates et al., 2015) package in R.

The model we started with included a random intercept for each participant, a fixed effect of trial (coded 0–2), and a random effect for trial. The random intercept was necessary because of the repeated measures research design. A fixed effect of trial was included based on previous research (Ishikawa and Montello, 2006; Courbois et al., 2013) and theoretical evidence (Montello, 1998) suggesting that route learning improves across trials. Barr et al. (2013) argue that maximizing the random effects structure allows for better generalization of the results. Therefore, we allowed the effect of trial to differ among participants as a random slope. By only allowing the effect of trial to vary among participants, we also ensured that all models compared have the same random effects structure. However, we also conducted the same model comparisons without using a random effect for trial. The results did not differ. A visual inspection of residual plots did not reveal any major deviations from the assumptions of our initial model.

We first tested the effect of route, which was dummy coded with the first route serving as the reference group. This effect was significant, indicating that participants made more errors when navigating route 1 (Table 2). We then tested the effect of group (DS and TD) on performance, which was dummy coded with DS as the reference group. This effect was not significant and was not included in the model. We also tested all two-way interactions and a three-way interaction, none of which were significant.

**TABLE 2:** Likelihood ratio tests for growth curve model comparisons.

	Model fitting criteria	Likelihood ratio tests			
Fixed effects in model	AIC	−2 log-likelihood	χ^2^	*df*	*p*-value
**Experiment 1**					
Trial (linear)	413.44	401.44	–	–	–
Trial (linear) + route	408.28	394.28	7.16	1	0.007
Trial (linear) + route + group	410.07	394.08	0.20	1	0.651
Trial (linear) × route + group	410.65	392.64	1.63	2	0.443
Trial (linear) + route × group	411.94	393.94	0.34	2	0.844
Trial (linear) × route × group	414.96	390.96	3.31	5	0.652
Trial (linear) + route + KBIT	409.83	393.84	0.44	1	0.506
Trial (quadratic) + route	407.64	391.64	2.63	1	0.105
**Experiment 2**					
Trial (linear)	152.56	140.56	–	–	–
Trial (linear) + group	149.78	135.78	4.77	1	0.029
Trial (linear) × group	146.86	130.86	4.93	1	0.026
Trial (quadratic) × group	142.21	122.21	8.65	2	0.013
KBIT + trial (quadratic) × group	144.10	122.10	0.10	1	0.748

Although the groups showed no mean difference on the KBIT-2 Matrices subtest, the DS group was more variable than the TD group, so we also tested the fixed effect of non-verbal ability as a potential covariate. One participant with DS was given a mean replacement score because of missing data. That test was not significant. Further, it could be that changes across trials are not linear, so an orthogonal polynomial for trial was created to test the quadratic trend. The quadratic trend was not significant. The results of the likelihood ratio tests are presented in Table 2.

The final model included fixed effects of trial and route. The estimates for the fixed effects of the final model are presented in Table 3. The individuals’ intercepts varied with an SD of 0.97 and the fixed effect of trial varied across individuals with a SD of 0.03. This indicates that little variability in route learning was explained by differences in the effect of trial. The SD of error not accounted for in the study was 1.52. These results suggest that learning errors decreased across trials and that this negative linear trend did not differ between the two routes or between the DS and TD groups. In addition, learning errors for Route 1 was higher than for Route 2; this difference was the same for all three trials and for both groups.

**TABLE 3:** Fixed effects for the growth curve models.

Experiment 1				Experiment 2			
Term	*b (SE)*	*t*	CI	Term	*b (SE)*	*t*	CI
Intercept	2.40 (0.33)	7.31	1.75–3.05	Intercept	4.63 (0.07)	64.31	4.49–4.77
Trial (linear)	−0.39 (0.20)	−1.96	−0.79–0.01	Trial (linear)	−0.11 (0.54)	−0.21	−1.17–0.87
Route 2	−0.89 (0.32)	−2.83	−1.57–0.26	Trial (quadratic)	−1.26 (0.49)	−2.58	−2.15 – −0.29
				TD group	−0.25 (0.11)	−2.32	−0.46 – −0.03
				Trial (linear) × TD group	−1.87 (0.81)	−2.31	−3.43– −0.23
				Trial (quadratic) × TD group	2.16 (0.72)	3.02	0.74–3.55

**CI = 95% bootstrap estimated confidence intervals*.*

#### Survey Learning

Correlations among survey learning tasks and the KBIT-2 are presented in Table 4. The only correlation that reached statistical significance (*r* = −0.592, *p* = 0.01) was the association between KBIT-2 scores and performance on the pointing task. However, all other correlations were in the expected direction. Specifically, each measure of survey learning was associated with better performance on all other measures of survey learning.

**TABLE 4:** Correlations among survey learning tasks in Experiment 1.

Measure	1	2	3
(1) KBIT			
(2) Shortcut	0.186		
(3) Map recognition	−0.022	−0.140	
(4) Not visible	−0.592*	−0.218	−0.147

KBIT, KBIT-2 matrices subtest raw score; not visible, direction of landmarks task for landmarks not visible on screen; *p = 0.01.

##### Shortcut navigation

The analysis of shortcut learning was conducted using a One-Way between-subjects ANOVA with Group (DS and TD) as the independent variable and number of segments traveled to reach the target as the dependent variable. The analysis indicated a significant effect of Group, *F*(1,19) = 8.894, *p* = 0.008, $\eta_{\text{p}}^{2}$ = 0.319. The participants with DS walked fewer segments to get to the target than did the TD children. The significant main effect remained following an analysis with KBIT scores as a covariate. In addition, inspection of the data indicated that four participants with DS and none of the TD participants took the shortest route to the target, confirming that the DS participants as a group actually performed better than the TD participants on the shortcut task.

##### Direction of landmarks

The analysis of the Direction of Landmarks performance was conducted using a Group × Visibility (landmark visible vs. landmark not visible) mixed effects ANOVA, with Visibility treated as a within-subjects variable. Because we were not interested in responses to individual trials, we averaged the errors across landmarks by Visibility. In this case, multiple analytical approaches would be appropriate. We settled on a mixed effects ANOVA, as opposed to the modeling approach used above, because the two tests would produce similar results, but the ANOVA focuses on the “average” effect across participants in each group. The main effect of Visibility was significant, *F*(1,20) = 5.35, *p* = 0.0314, $\eta_{\text{p}}^{2}$ = 0.211. However, neither the main effect of Group, *F*(1,20) = 1.218, *p* = 0.283, $\eta_{\text{p}}^{2}$ = 0.057, nor the two-way interaction, *F*(1,20) = 1.255, *p* = 0.276, $\eta_{\text{p}}^{2}$ = 0.059, was significant. Both groups located the visible landmarks more easily than they did the not visible landmarks (7.5 degrees vs. 9.9 degrees divergence, respectively). However, the groups did not differ from each other. The performance of both groups was also significantly better than would be expected if they were responding in random fashion: *t*(20) = 6.33, *p* < 0.001, *d* = 1.06 for the visible and *t*(20) = 4.84, *p* < 0.001, *d* = 1.42 for the not visible landmarks.

##### Map recognition

The analysis of Map Recognition performance was conducted using two Chi Square analyses. First, we conducted a Test of Independence to determine whether the groups differed in their ability to select the most accurate map representation. As expected from visual inspection of the data, the groups did not differ, χ^2^ (1) = 0.0063, *p* = 0.94. Second, we conducted a Goodness of Fit test to determine if the performance of our participants (both groups combined) differed from chance performance (i.e., choosing the correct response at a rate of greater than 20%). The results of this analysis yield a significant effect, χ^2^(1) = 12.68, *p* < 0.0004. Hence, overall the participants performed above chance on the map recognition test, but groups did not differ.

### Discussion

The results of the Experiment 1 indicated that the participants with DS performed no worse on any of our measures of route or survey learning than did the TD children. This was a little surprising given that Davis et al. (2014) found the people with DS performed below expected levels on route learning relative to mixed ID participants and to TD children. However, in our current study the participants with DS committed numerically fewer errors on several individual trials across both routes and traveled significantly fewer segments to reach the target in the shortcut task.

One reason for this discrepancy is likely that we created environments that were easier for all participants to learn so we could focus on survey knowledge. This provided participants with an optimal opportunity to demonstrate survey knowledge following the learning of the routes. If they were unable to learn the routes, then they would not have been able to access very much survey knowledge. It would be interesting to identify the mechanisms of wayfinding responsible for magnifying group differences as environments get more complex. There are certainly a number of plausible personal factors (e.g., spatial working memory, sequence memory, route integration, etc.). Nevertheless, our data indicate that survey learning need not be a deficiency in DS relative to TD children under optimal learning conditions using simple and predictable routes. Purser et al. (2015) also found that non-verbal abilities could explain many of the differences between people with DS and TD children on numerous spatial tasks. It may be that our choice to match on non-verbal ability limited the likelihood of observing differences in performance on our wayfinding measures. The choice of matching criteria is always important. In this case, we wanted to know if wayfinding skills present a unique problem for people with DS relative to other non-verbal abilities. At least for the conditions of Experiment 1, where we used a small, predictable environment, the answer appears to be no. Further, this was true for multiple measures of survey knowledge.

One limitation of our results may be that the sample size was relatively small. This is especially problematic when assessing the correlations among survey learning tasks. All of the correlations were in the expected direction, but few reached statistical significance. We feel confident in concluding that the tasks did indeed measure the same underlying construct, survey knowledge, even if the correlations were largely inconclusive. It could also be argued that there were few significant differences between groups on the wayfinding tasks because of a lack of statistical power. However, given the current results, it is not clear that adding participants would increase the possibility of obtaining a significant result in favor of the TD children. In fact, the performance of the participants with DS was numerically better than the TD participants on several measures. That is, the difference was in the opposite direction as is typically found. Although the participants with DS performed better than the TD participants on the shortcut learning task, there was insufficient evidence to identify group differences on any of the other tasks. For this reason, we are most comfortable in concluding that our participants with DS performed at least as well on our measure of route learning and most measures of survey knowledge as the TD participants. However, it is interesting that the participants with DS outperformed the TD participants when navigating using a shortcut, at least under optimal learning conditions. Still, it will be necessary to replicate these results using multiple samples and methods for verification, and to identify the variables that may have led to this particular result.

## Experiment 2

Experiment 2 compared individuals with DS to TD children on measures of survey learning and landmark recognition over multiple exposures to the environment without first requiring them to learn individual routes. The groups of participants were approximately matched on non-verbal ability using the KBIT-2 Matrices subtest (Kaufman and Kaufman, 2004). An experimenter navigated a circuitous path through a virtual environment five times while participants watched. All presentations began and ended at a specific target (i.e., the only door in an office building). After the first, third, and fifth presentation of the environment, participants were tasked with finding the shortest path possible to a known target. Distance traveled at each trial served as a measure of survey learning. Consistent with past research (Farran et al., 2015; see also Courbois et al., 2013), we expected that participants with DS would travel a longer distance to find the target than would the TD participants. We also expected that participants with DS would recognize fewer landmarks.

### Method

#### Participants

The participant groups consisted of 20 adolescents/young adults with DS and 17 TD children. Participants were paid $5.00 for completing the tasks. The participants with DS were recruited from the University of Alabama Intellectual Disabilities Participant Registry and from local service providers. Different service providers were recruited to limit targeting the same participants in each study. Nevertheless, three participants with DS were included in both studies. Because the time between studies was greater than 8 months for these participants and because the studies involved very different environments and procedures, we determined that practice effects across studies would be minimal. Further, analyses conducted with and without the three overlapping participants yielded results that were largely identical. Therefore, except as noted only the analyses including all of the participants are presented below. Parents or guardians confirmed a diagnosis of DS. The TD participants were recruited from local preschool programs. The mean age of the participants with DS was 19 years and 8 months (*SD* = 37 months) and for the TD children was 5 years and 5 months (*SD* = 11 months). The groups were poorly matched on gender (DS: 4 females; TD: 13 females). Two TD participants chose not to complete the landmark recall task, resulting in 15 TD participants for that test.

#### Measures

##### Kaufman brief intelligence test-2 – matrices

All participants completed the Matrices subtest of the KBIT-2.

##### Survey-learning task

**FIGURE 6:**
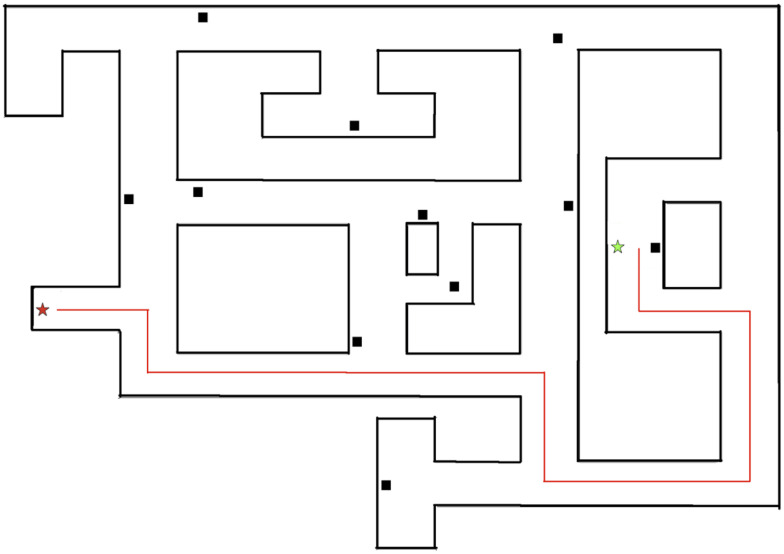
Overview of Experiment 2 environment. The target is depicted by a red star and the starting point for the shortcut trial is depicted by the green star. The fastest possible path to the target is depicted by the red line.

Participants were tasked with finding the shortest path to a target in a virtual environment (Figure 6). The environment was constructed using the FPSCREATOR software. The environment was modeled after a typical office building and contained nine thematically appropriate landmarks. There were nine unique landmarks, including a door (the target of the short cut task), small blue cabinet, conference table, computer workstation, large black cabinet, painting on wall, water cooler, flip chart, desk, and chair. In addition to these nine unique landmarks, there were three identical couches that appeared. This was done to better mimic real-world environments, in which not every landmark is unique. The specific environment was chosen after pilot testing with typical adults and young adults with intellectual disability but not Down syndrome demonstrated that the environment was unlikely to result in ceiling or floor effects. All computer tasks in Experiment 2 were completed on an Acer Aspire 5253 laptop with a 15.6″ monitor and a screen resolution of 1,280 pixels × 1,024 pixels. All participants sat approximately 60–75 cm from the display.

All participants were able to familiarize themselves with the navigational controls prior to beginning the task. The experimenter presented the environment by navigating along a circuitous path that began and ended at the same landmark in the environment. The participant was placed in the environment at a location along the path taken by the experimenter (see Figure 6) and tasked with finding the shortest possible path to the target (Trial 1). Once the participant found the door, the experimenter presented the environment two additional times and participants were again placed at a starting point and traversed to the target (Trial 2). The experimenter again presented the environment two times, followed by the participants’ task of finding the shortest path (Trial 3).

The environment was constructed by connecting a discrete number of equal sized blocks, which appeared to participants as a continuous environment. The total distance traveled was measured by counting the number of blocks traversed on each trial. The shortest possible path to the target was 47 blocks. If participants successfully navigated the shortest path to the target on the first or second trial, no additional trials were completed.

##### Landmark recognition task

Following the survey learning task, participants completed a measure of landmark recognition. Participants were shown two potential landmarks side-by-side. One was a landmark from the environment and the other was a similar object that did not appear in the environment. All pictures were shown from the same perspective that the object would have been viewed during the experimenter’s presentations of the environment. Participants were asked to point to the object that they had seen in the virtual environment. There were nine unique trials presented in random order for each participant. The dependent variable was the total number of landmarks correctly recognized.

#### Procedure

Parents or guardians gave consent and all participants gave assent. Participants completed the KBIT-2 Matrices subtest, then they were presented with the virtual environment. To navigate the environment, participants used a mouse to look around and the ‘w’ or ‘up-arrow’ key to move forward. To become better acquainted with the controls, a researcher demonstrated how to look in each direction and move forward, then the participant mimicked the behaviors of the researcher. The participants were then tasked with navigating a short ‘L’ shape, then turning around and going back without researcher assistance.

After explaining the instructions for the task, an experimenter presented the environment to participants by navigating a circuitous path along the environment, pausing to look at and naming each landmark in the environment. Further, the experimenter paused at each choice point and looked both directions while verbalizing the action to participants (e.g., “I am going to stop and look right, then look left. I am going to go this way.”). The experimenter’s path began and ended at the only door in the environment, which served as the target that participants were to find. The presentation of the environment was again experimenter controlled to allow the e experimenter to refocus the participant’s attention as needed during the presentation. The shortcut task was then completed by participants. The experimenter traversed the environment two additional times, then the participants completed the second trial of the shortcut task. The experimenter traversed the environment twice more and the participant completed the third trial of the shortcut task. If a participant found the target in the fewest number of blocks, then no more trials were completed. Following the maximum number of three trials, the landmark recognition task was presented. The total procedure took approximately 45–60 min.

### Results

#### KBIT-2

Descriptive statistics for the primary variables are presented in Table 5. A preliminary analysis of KBIT-2 Matrices Subtest scores revealed the groups were not significantly different on measured non-verbal ability [*F*(1,35) = 0.62, *p* = 0.436, $\eta_{\text{p}}^{2}$ = 0.017]. Qualitatively, data from the shortcut learning task revealed that one participant with DS reached the target in the shortest possible distance, and did so on the first trial. In addition, six TD participants found the target in the shortest possible path, with two participants doing so on the second trial and four on the third trial. Quantitative analyses were conducted on distance traveled to evaluate changes in ability locate the target location as a function of increased exposure to the environment.

**TABLE 5:** Descriptive statistics for Experiment 2 (means with standard deviations in parentheses).

Group	KBIT	Trial 1	Trial 2	Trial 3	Landmarks
DS (*n* = 20)	14.1 (5.1)	108.9 (59.2)	137.4 (69.6)	105.2 (56.7)	6.1 (1.7)
TD (*n* = 17)	15.3 (4.5)	118.1 (54.3)	76.5 (36.2)	73.7 (31.5)	7.5 (0.9)

**KBIT, KBIT-2 matrices subtest raw score; Trial, distance traveled in the survey learning task; Landmarks, landmarks successfully recognized*.*

#### Survey Learning

Growth curve analysis was used to analyze the distance (number of blocks) taken to find the target over the course of the three trials in the survey learning task. We used the same modeling approach used in Experiment 1. Specifically, we started with a basic model and then sequentially added fixed effects and compared the models. The model we started with included a random intercept for each participant, a fixed effect of trial (coded 0–2), and a random effect for trial. Our reasoning for starting with this model is the same as in Experiment 1. We conducted the model comparisons without a random effect of trial and the results remained consistent. A visual inspection of residual plots revealed deviations from the assumption of normality in our initial model. Therefore, a natural log transformation was used on the distance variable. A visual inspection of the new residual plots did not reveal major deviations from homoscedasticity or normality. The model statistics presented below are for the transformed data.

We first tested the effect of group (DS and TD) on performance, which was dummy coded with DS as the reference group. This effect was significant, meaning that there was a difference in performance between the two groups, and was included in the model (Table 2). We then tested a trial by group interaction, which was also significant. Specifically, the TD participants improved their performance across trials, but the participants with DS showed little change. However, we had no reason to assume that the change across trials should be linear. An orthogonal polynomial for trial was created to test the quadratic trend and a trial by group interaction was tested again. This model was significant, indicating that the quadratic trend was a better fit to the data. Finally, although the groups showed no mean difference on KBIT-2 Matrices subtest, we tested the fixed effect of non-verbal ability as a potential covariate. That test was not significant and not included in our final model. The results of the likelihood ratio tests are presented in Table 2.

**FIGURE 7:**
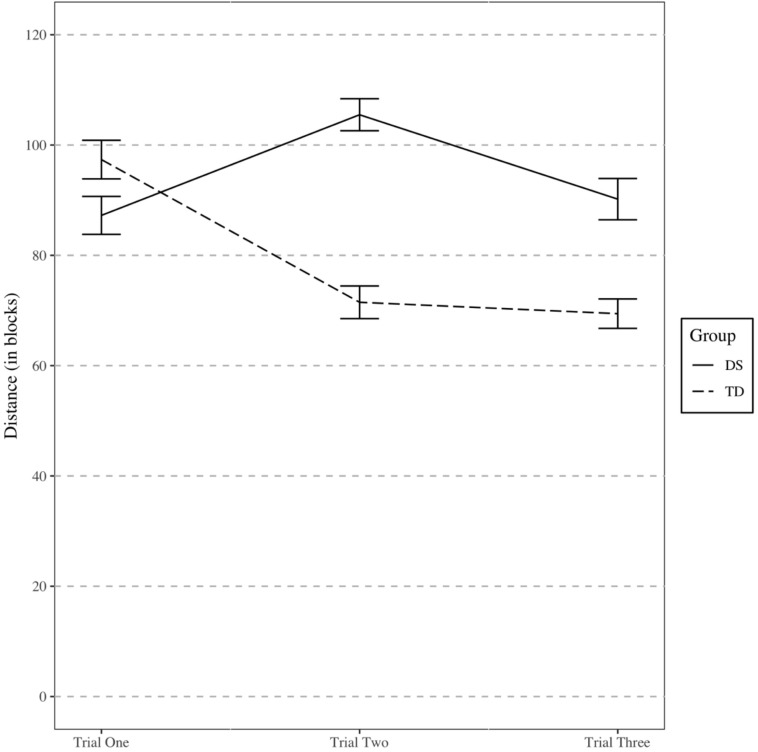
Distance traveled during Experiment 2 shortcut task. Error bars represent one standard error.

The final model included an orthogonal quadratic effect of trial, group, and an interaction between the terms as fixed effects. The estimates for the fixed effects of the final model are presented in Table 3 and a graph of the interaction is presented in Figure 7. The individuals’ intercepts varied with an SD of 0.29 and the fixed effect of trial varied across individuals with a SD of 0.13. The SD of error not accounted for in the study was 0.36.

When we reanalyzed the data without the three overlapping participants from Experiment 1, the results were largely similar but contained one difference. Specifically, trial by group interaction was no longer statistically significant (*p* = 0.104), though the *AIC* did suggest an improved model fit when the interaction term was included. The quadratic trial by group interaction did provide a statistically significant (*p* = 0.008) improvement to model fit compared to a model without an interaction, though, so we retained the same model in both sets of data.

#### Landmark Recognition

The data for number of landmarks correctly recognized did not meet the normality or homogeneity of variance assumptions for a parametric test. Therefore, a non-parametric test was used. The median number of landmarks recalled in the DS (median = 6, *n* = 20) and TD (median = 8, *n* = 15) groups differed significantly (Mann–Whitney *U* = 69, *p* = 0.006, Cliff’s *d* = −0.588).

### Discussion

The results from Experiment 2 are consistent with the hypotheses and previous research. Specifically, participants with DS were similarly able as TD children to find the shortest path to a target after their first exposure to an environment. On subsequent trials, though, the participants with DS were less able to find the target as efficiently as the TD children. Further, participants with DS recognized fewer landmarks. Hence, our results are consistent with previous research in demonstrating a relative weakness in landmark memory (Courbois et al., 2013; Davis et al., 2014) and survey knowledge (Farran et al., 2015) in individuals with DS. Further, they demonstrate that survey knowledge develops more slowly over successive exposures to the environment for individuals with DS relative to TD children.

Interestingly, on the first trial, participants with DS did not travel a longer distance to find the target than TD. However, TD participants generally exhibited improved performance across repeated trials, whereas participants with DS generally did not. This resulted in better performance for TD participants in trials two and three. Thus, the pattern of results is suggestive of an inefficiency in accumulating survey knowledge through repeated exposures to an environment or in effectively using a survey representation to take a novel shortcut. This is consistent with the findings of Courbois et al. (2013), who found that participants with DS did not differ significantly from TD children in their first trial on a shortcut task. Unlike the current results, though, Courbois et al. (2013) also did not find a significant difference in the final trial whereas the current study did. This difference may be due to the relatively low number of participants that participated in that part of their study (*n* = 16).

A quadratic trend was found to match the data better than a linear trend. For the participants with DS, performance was better on the first trial than on the second trial. The descriptive statistics revealed much more variation in performance for participants with DS, beginning after the first trial. This could explain the pattern of getting worse on trial 2. The quadratic trend was also driven by the TD participants, who tended to much more learning from trial 1 to trial 2 than from trial 2 to trial 3. This pattern could indicate that these participants learned most of what they were able to learn within the first few exposures to a novel environment. Alternatively, it could be that the TD children did not understand the task in trial 1, but better understood what was asked of them by trial 2.

The current findings are consistent with an explanation that abnormal development of the hippocampal regions is associated with the observed weakness in survey learning. Specifically, the hippocampus (Maguire et al., 1998) and medial entorhinal cortex (McNaughton et al., 2006) have been associated with the formation of survey representations (see Burgess, 2008 for a review). The hippocampal region has been identified as being smaller relative to overall brain size in individuals with DS (Aylward et al., 1999; Pinter et al., 2001a) compared to TD individuals. Pennington et al. (2003) showed that individuals with DS perform less well on measures associated with hippocampal functioning than would be expected given their cognitive phenotype, suggesting that the neurodevelopmental abnormalities are accompanied by specific behavioral weaknesses.

We also found that participants with DS correctly recognized fewer landmarks than TD children. This finding is consistent with Courbois et al. (2013) and Davis et al. (2014). Individuals with DS seem to be less selective when attending to landmarks, resulting in them disproportionately focusing on fewer landmarks located at choice points (Davis et al., 2014). In the current study, there was no prescribed path that participants had to take. However, the individuals with DS may have not relied as much on information from landmarks, which may have made their performance less consistent across trials. Because we only measured landmark recognition after participants finished the shortcut task, we are not able to conclusively determine the relationship between landmark recognition and online survey learning. However, it is reasonable to believe that such a relationship exists and that being able to recognize landmarks from different perspectives plays an important role in identifying alternative routes to a target location (Karimpur et al., 2016).

## General Discussion

In two experiments, we observed similarities and differences in the acquisition of survey knowledge by people with DS and TD children with whom they were approximately matched on non-verbal MA.

In Experiment 1, we were able to identify a number of similarities in what types of survey information was acquired following explicit route learning in a simple environment. After learning two overlapping routes in the environment, the participants with DS exhibited learning that was at least as high as the TD participants on three different measures of survey knowledge: shortcut performance, identifying the direction of unseen landmarks from a designated location in the environment, and selecting a map of a bird’s eye view of the environment. Hence, it appears that once the two routes for the environment were learned, the majority of the participants with DS were able to exhibit some general knowledge of the overall environmental layout. Further, the degree of knowledge exhibited by these participants was similar to what may be expected based on their level of general cognitive performance. Taken together, the two experiments suggest that even though the acquisition of survey learning may take more time when people with DS are simply exploring the environment, under conditions of explicit learning they can acquire at least some survey knowledge through the learning of overlapping routes. This is consistent with Courbois et al. (2013) who found that some participants with DS could identify a shortcut after learning routes in a similar simple environment.

Although several of the participants with DS could exhibit shortcut learning, their overall performance was likely much less than that of similar CA participants without intellectual disability (see Courbois et al., 2013). That result and our results still suggests important limitations on everyday wayfinding and navigation for people with DS. Only a third of our participants actually found the shortest route in the shortcut task, and only about half were able to identify a bird’s eye view of the environment. Even performing at a level roughly the same as the MA matched TD children may not be particularly consequential. Indeed, the TD children evaluated in our study are likely just beginning to show the learning of survey knowledge themselves. For example, Cousins et al. (1983) found that tests of survey knowledge that involved estimating the positions of landmarks was late developing compared to route and landmark learning in a test of 7, 10, and 13-year-old children with only a portion of the oldest group effectively demonstrating this knowledge. However, it does appear that some survey knowledge can be learned by children as young as 4-years-old (e.g., Hazen et al., 1978; Huttenlocher et al., 2008). For example, Huttenlocher et al. (2008) found that children between 3 and 4 years of age were able to use a scale model to find an object in a larger scale environment. Hence, it is likely that both the participants with DS and TD children were exhibiting rudimentary survey knowledge at best. Further, while it may be expected that the TD children will gradually acquire additional abilities to represent survey knowledge with increasing CA and experience with environmental learning, the same outcome cannot be assumed for people with DS. They have already had much more general experience with the environment than have the TD children and are still exhibiting beginning level survey knowledge.

In Experiment 2, we found an important difference in that it took longer for our participants with DS to acquire survey knowledge relative to the TD children when they were exposed to the environment over a series of trials. We patterned this task to mimic being walked around the neighborhood and incidentally acquiring knowledge of the overall environment. The results clearly suggested that our participants with DS had more difficulty learning the environment under these conditions than did the TD children. It may be that people with DS are less likely to focus on information relevant to navigation unless explicitly told to do so when they experience an environment. This was true in spite of the fact that the participants were aware that a portion of the task was to navigate the environment after being led around. However, because we stopped the experiment after 5 exposures, it is not clear whether or not the participants with DS might eventually achieve a level of performance similar to that of the TD children. Further, we acknowledge the possibility that the complexity of the route may have adversely affected the participants with DS more than it did the TD children. We would need to replicate these results across different levels of complexity and different numbers of exposure to determine the generality of this conclusion.

With respect to survey learning in particular, we think the possible discrepancy between Experiment 1 and Experiment 2 may be more apparent than real. In Experiment 2, the focus was on how rapidly survey learning takes place for persons with DS. In Experiment 1, we asked about the kind of survey knowledge that could be accessed following learning under optimal conditions. Hence, a different pattern of results may be expected. Indeed, Experiment 1 suggests that survey knowledge of a learned environment may be similar for participants with DS and TD children. Experiment 2 suggests that, at least for longer and less predictable environments, it may take more time for the participants with DS to acquire that knowledge. One real possibility is that the larger and more unpredictable environment involved more visual processing resources to be completed than did the small environment of Experiment 1. Research has clearly demonstrated that visual working memory is important to constructing survey representations of the environment (see for example, Wen et al., 2013; Piccardi et al., 2019). Recent research has indicated that people with DS may have some weaknesses in some aspects of visual processing involving spatial memory and visuoconstructive tasks (Fidler, 2005). Hence, we might expect greater differences in tasks that require a greater use of these processes as in Experiment 2.

A big question that remains is whether it is possible to build on existing skills of survey knowledge acquisition as identified in Experiment 1 to promote better survey learning in people with DS. As noted in the introduction, there is considerable overlap between brain abnormalities reported in DS and those regions of the brain known to support wayfinding activities (e.g., Maguire et al., 1998; Aylward et al., 1999; Pinter et al., 2001a). To what degree do these differences constrain environmental learning in DS? One interesting feature of spatial ability performance is that spatial abilities appear to be relatively malleable across a range of ages and ability levels in persons without DS. In a meta-analysis of over 200 studies, Uttal et al. (2013) found that training and experience can produce positive and lasting effects in adults and children. For example, video game activities (e.g., Green and Bavelier, 2003; Spence and Feng, 2010), puzzle completion (Levine et al., 2012), and building activities (Coxon, 2012) promote the development of spatial abilities such as mental rotation, perspective taking, and spatial visualization in TD children and adults. To the extent that spatial abilities that are involved in acquiring survey knowledge of the environment are malleable in people with DS, then it may be possible to promote the acquisition of survey knowledge during environmental learning by people with DS. It is important for future research to explore this possibility.

## Conclusion

Our conclusions must be considered in the context of some experimental limitations. We used TD children matched on non-verbal ability as a comparison group to evaluate the acquisition of environmental knowledge in people with DS. However, there are many differences between young adults with DS and TD children beyond just those directly associated with the DS genotype. For example, our participants with DS may have a wider range of experiences navigating unfamiliar environments, which may lead to the developing more efficient heuristics. These heuristics may work relatively well in the simple environment presented in Experiment 1, but not in the irregular and complex environment presented in Experiment 2. There are other differences between these young adults with DS and TD children that may also impact our results. It is important for future studies to use alternative comparison groups, such as other adults with intellectual disability, to help rule out certain confounds. Another limitation is that the study was conducted in a virtual reality environment. Navigation in the real world provides additional aids that are not available in virtual environments, such as proprioception (Waller et al., 2004), peripheral visual cues (Alfano and Michel, 1990), and other sensory cues (Chrastil and Warren, 2013). Many studies have shown an approximate equivalence between real world and virtual environmental learning (e.g., Coutrot et al., 2019), including for atypical populations (Claessen et al., 2016). However, it is still an open question whether individuals with DS have specific difficulties navigating in virtual environments.

## Data Availability Statement

The datasets generated for this study are available on request to the corresponding author.

## Ethics Statement

The studies involving human participants were reviewed and approved by The University of Alabama IRB. Written informed consent to participate in this study was provided by the participants’ legal guardian/next of kin.

## Author Contributions

EM, ZH, FC, BR, and YY contributed to design of research. EM, ZH, FC, and TR contributed to stimulus preparation and final execution of procedure. EM, ZH, FC, BR, YY, and TR contributed to data analysis and interpretation. EM, ZH, FC, BR, YY, and AR contributed to the final production of the manuscript. All authors contributed to the article and approved the submitted version.

## Conflict of Interest

The authors declare that the research was conducted in the absence of any commercial or financial relationships that could be construed as a potential conflict of interest.
